# Supplementary material for: CD69 Signaling in Eosinophils Induces IL-10 Production and Apoptosis via the Erk1/2 and JNK Pathways, Respectively
Source: Biomolecules. 2024 Mar 18;14(3):360. doi: 10.3390/biom14030360 (PMC10968075; doi:10.3390/biom14030360)
Supplement: Supplementary file 1 [file biomolecules-14-00360-s001.zip › biomolecules-2817845-supplementary.pdf]

ONLINE SUPPORTING INFORMATION

CD69 Signaling in Eosinophils Induces IL-10 Production and Apoptosis via the Erk1/2 and JNK Pathways, Respectively

AUTHORS: Dan Van Bui, Linh Manh Nguyen, Akira Kanda, Hanh Hong Chu, Nhi Kieu Thi Le, Yasutaka Yun, Yoshiki Kobayashi, Kensuke Suzuki, Akitoshi Mitani, Akihiro Shimamura, Kenta Fukui, Shunsuke Sawada, David Dombrowicz and Hiroshi Iwai

SUPPLEMENTARY METHODS

Lung cell preparation

Mouse lungs were harvested and digested in a collagenase–DNase mixture from the Lung Dissociation Kit (Miltenyi, Bergisch Gladbach, Germany), according to the manufacturer's instructions. A single cell suspension was obtained by passing the lung cells through a 70-µm cell strainer (Thermo Fisher Scientific, MA, USA).

Eosinophil purification

Splenocytes were incubated with biotin-conjugated anti-CD19, anti-CD90.2, and anti-CD8α antibodies (BD Pharmingen, CA, USA), followed by incubation with anti-biotin–conjugated magnetic beads (Miltenyi, Bergisch Gladbach, Germany). Purity and survival rates were greater than 90%. The FACSAria III (BD Biosciences, NJ, USA) cell sorter was used to isolate eosinophils for quantitative polymerase chain reaction examination. Eosinophils were gated as CD19-CD90.2-Gr-1intSiglec-F+, and the purity of eosinophils was >99%.

RNA isolation

RNA from purified eosinophils was extracted using a RNeasy Mini Kit (Qiagen, Hilden, Germany). PrimeScript RT Master Mix (Takara, Shiga, Japan) was used to reverse transcribe the extracted RNA to the first-strand cDNA.

Flow cytometric analysis and CD69^{+}-Eosinophils sorting

To analyze CD69 expression in ovalbumin (OVA)-induced asthma mice, single-cell suspension from lung tissues was stained with the monoclonal antibodies CD45, CD11b, Gr-1, Siglec-F (BD Biosciences, NJ, USA), and CD69 (Miltenyi, Bergisch Gladbach, Germany) and/or corresponding isotype IgG for 30 min at 4°C. FACS Canto II flow cytometer and Aria III (BD Biosciences, NJ, USA) were used to measure the samples. CD69^{+}-Eosinophils from healthy and asthmatic mouse lung and spleen were sorted by identification as CD45^{+}CD11b^{Hi}Gr-1^{Int}Siglec-F^{Hi}CD69^{+}and CD90.2^{-}CD19^{-}Gr-1^{Int}Siglec-F^{Hi}CD69^{+}, respectively. FlowJo software (BD Biosciences, NJ, USA) was used to analyze the data obtained.

Immunofluorescence staining

Section samples from purified eosinophils or frozen lung tissues were incubated with anti-mouse SiglecF (1 μg/mL; eBioscience, CA, USA), followed by anti-mouse CD69 (2.5 μg/mL; Santa Cruz Biotechnology, TX, USA) overnight; thereafter, the samples were incubated with 1/200 secondary antibodies for 1 h at room temperature (RT). Hoechst (2.0 μg/mL; Dojindo, MD, USA) was used to perform counterstaining for 10 min at RT. The FV3000 confocal microscopes (Olympus, Tokyo, Japan) were used to measure these section samples, and FV10-ASW FluoView software (Olympus, Tokyo, Japan) was used to analyze them.

Western blot

M-PER lysing buffer (Thermo Fisher Scientific, MA, USA) containing phosphatase/protease inhibitors (Cell Signaling Technology, MA, USA) was used to prepare the whole-cell protein extracts. A total of 25-μg protein was loaded onto 7.5% polyacrylamide gels (Bio-Rad, CA, USA) and then transferred to polyvinylidene fluoride membrane (Bio-Rad, CA, USA). Membranes were blocked using the Intercept Blocking Buffer (LI-COR, NE, USA) for 1 h at RT and stained with antibodies for total or phosphorylated Erk1/2, STAT5, Jak3, JNK (Cell Signaling Technology, MA, USA), and β-actin (Santa Cruz Biotechnology, TX, USA) overnight at 4°C. Odyssey software 3.0 (LI-COR, NE, USA) was used to quantify the proteins, and Image Studio Lite software 5.2.5 (LI-COR, NE, USA) was used to analyze the images.

SUPPLEMENTARY TABLE

Table S1. Primer List

	Forward	Reverse
GAPDH	5′-TGCCCAGAACATCATCCCTG-3′	5′-TCAGATCCACGACGGACACA-3′
GATA3	5′-GCCTGCGGACTCTACCATAA-3′	5′-CATTAGCGTTCCTCCTCCAG-3′
IFNγ	5′-ACTCAAGTGGCATAGATGTGG-3′	5′-AAGACTTCAAAGAGTCTGAGGTAG -3′
IL-4	5′-ACACCACAGAGAGTGAGCTCG-3′	5′-TGCAGCTCCATGAGAACACTAG-3′
IL-10	5′-ATTTGAATTCCCTGGGTGAGAAG-3′	5′-CACAGGGGAGAAATCGATGACA-3′
IL-13	5′-AAAACTGCAGCAAGACCGTG-3′	5′-CCAGAGCCCACTGCTTCAAT-3′
IL-17A	5′-TACAGTGAAGGCAGCAGCGATC-3′	5′-ACATTCTGGAGGAAGTCCTTGG-3′
RORγ	5′-ACTGAGGCCATTCAGTATGTGG-3′	5′-TGCACATTCTGACTAGGACGAC-3′
T-bet	5′-CCCACAAGCCATTACAGGATGT-3′	5′-TGCCTTCTGCCTTTCCACAC-3′
TGF-β	5′-ACCATCCATGACATGAACCGG-3′	5′-AGCAGTTCTTCTCTGTGGAGC-3′

SUPPLEMENTARY FIGURES

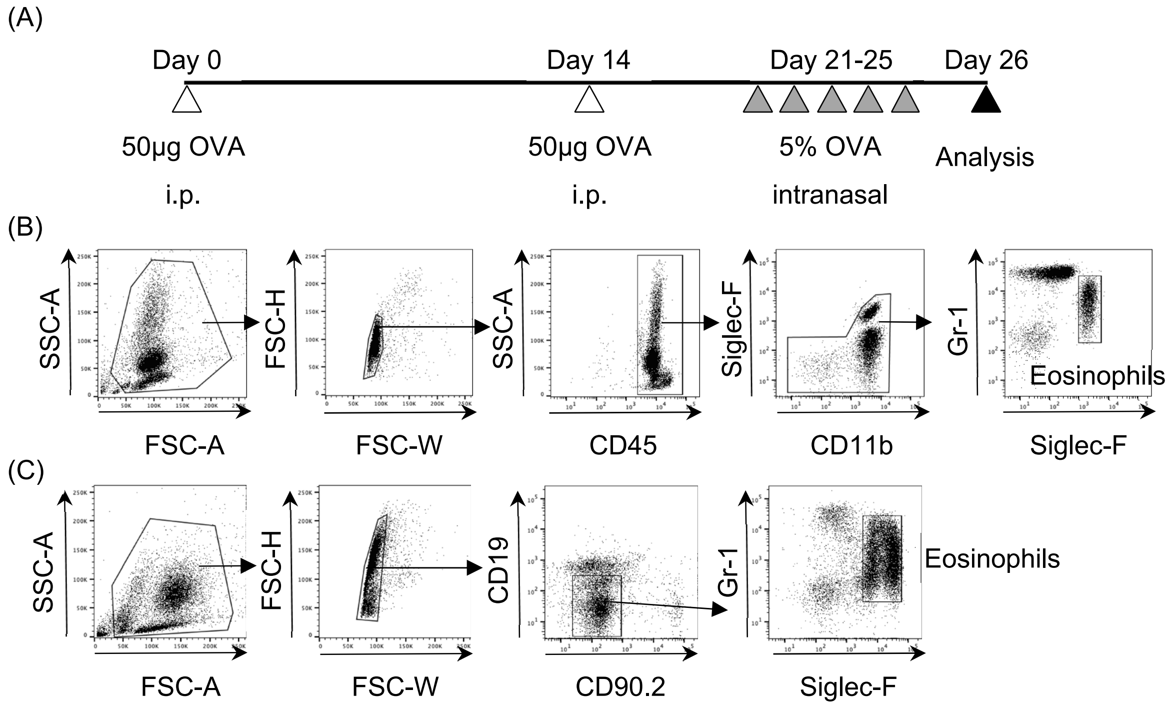

Figure S1. Schema of experimental protocol and eosinophil isolation

(A) Experimental design of OVA-induced asthma mice model. (B) and (C) Eosinophil gate strategy using flow cytometry analysis. Eosinophils from healthy and asthmatic mouse lung and spleen were identified as $CD45^{+}CD11b^{Hi}Gr\text{-}1^{Int}Siglec\text{-}F^{Hi}$ and $CD90.2^{-}CD19^{-}Gr\text{-}1^{Int}Siglec\text{-}F^{Hi}$, respectively. i.p.; indicates intraperitoneal.

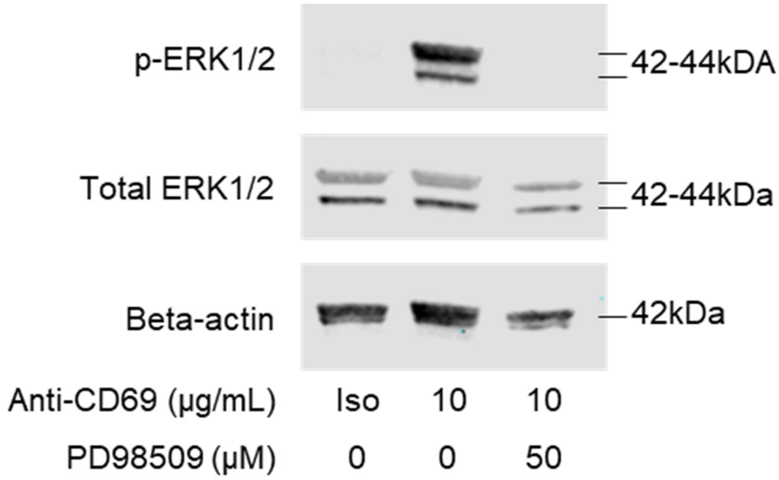

Figure S2. Phosphorylation of Erk1/2 following CD69 crosslinking on eosinophils in the presence of PD98509

Western blot analysis for phosphorylated and total Erk1/2 was shown. PBS or 50 µM PD98509 was incubated for 1 h prior to CD69 crosslinking on eosinophils purified from IL-5Tg mice for 30 min.

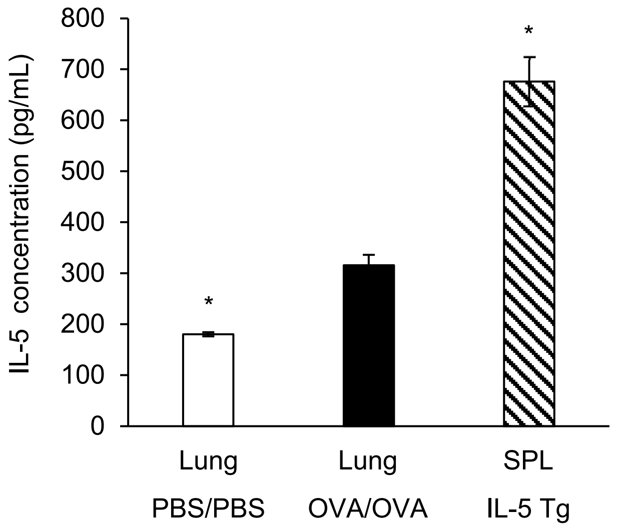

Figure S3. IL-5 protein in the supernatant

The IL-5 concentration in supernatants obtained from lung cells of healthy mice (PBS/PBS), lung cells of OVA-induced asthma mice (OVA/OVA), and splenocytes (2 million cells/250 μL/well) from IL-5Tg mice for 24 h incubation was measured using an ELISA kit. Data are expressed as mean ± SEM (n = 6 for each group). * indicates a significant difference compared with healthy mice ($p < 0.05$). SPL; spleen.
