# Supplementary material for: CD69 Signaling in Eosinophils Induces IL-10 Production and Apoptosis via the Erk1/2 and JNK Pathways, Respectively
Source: Biomolecules. 2024 Mar 18;14(3):360. doi: 10.3390/biom14030360 (PMC10968075; doi:10.3390/biom14030360)
Supplement: Supplementary file 1 [file biomolecules-14-00360-s001.zip › Western blot raw.pdf]

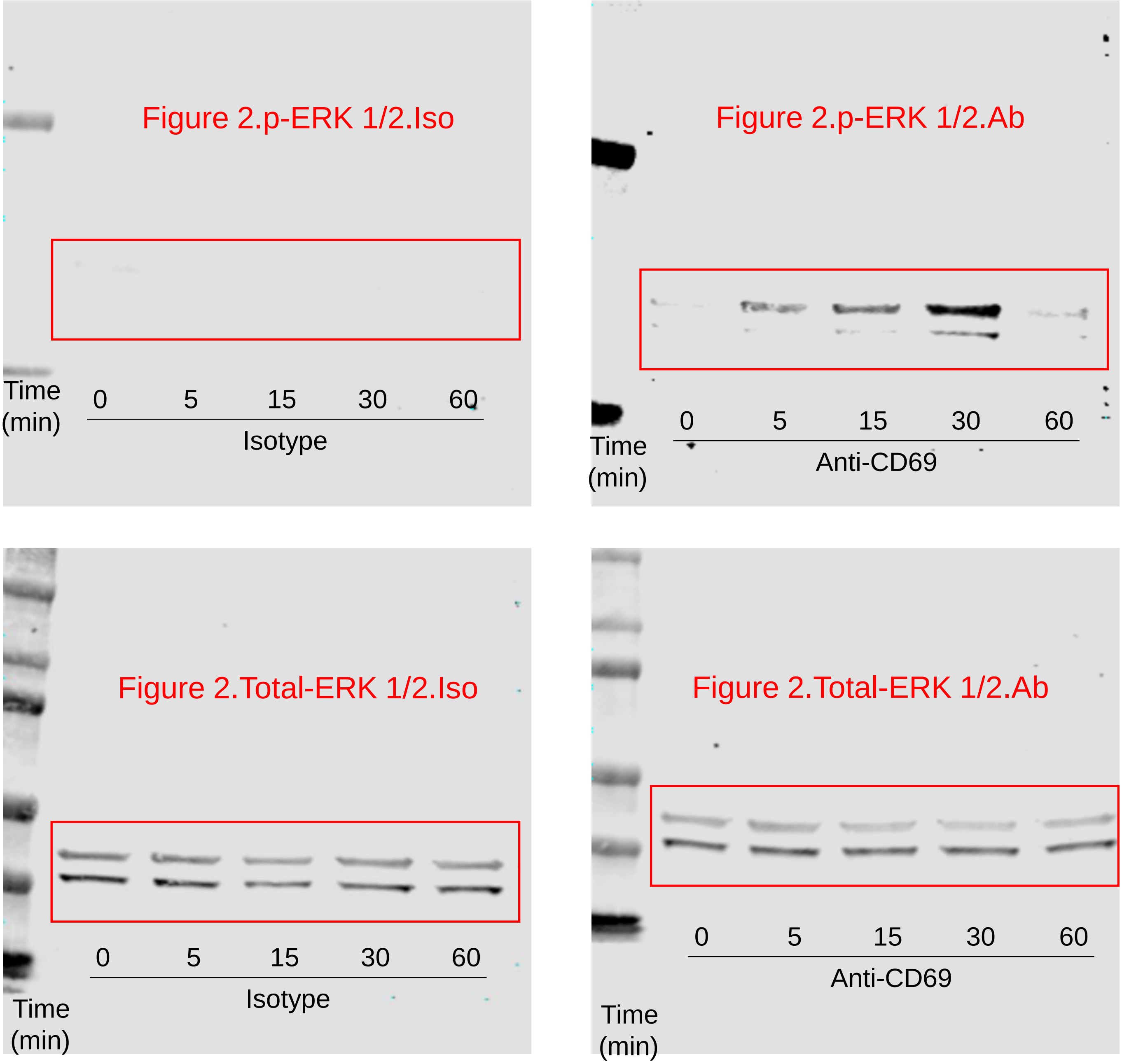

Figure 2.Western blot raw

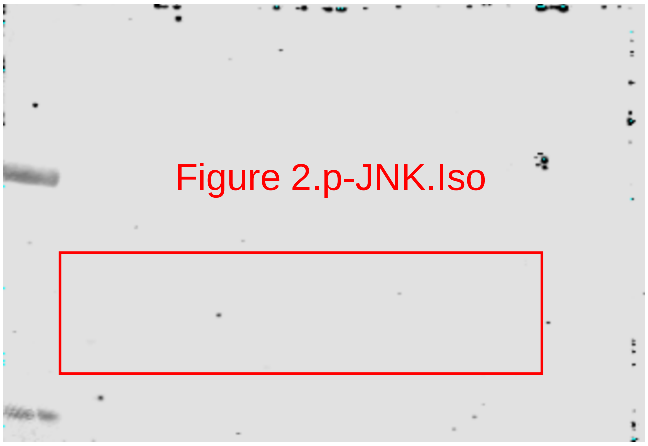

Time (min) 0 5 15 30 60

Isotype

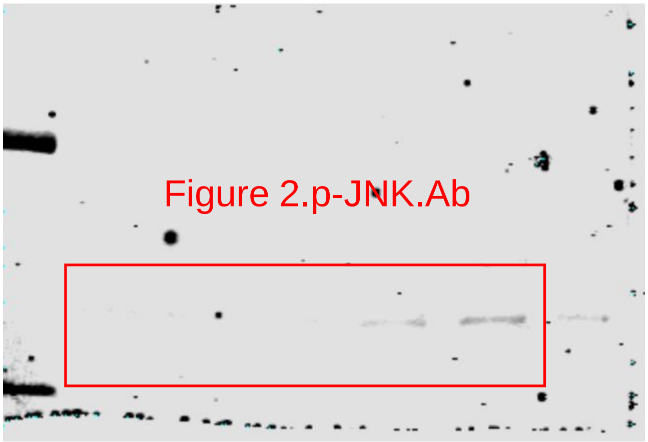

Time (min) 0 5 15 30 60

Anti-CD69

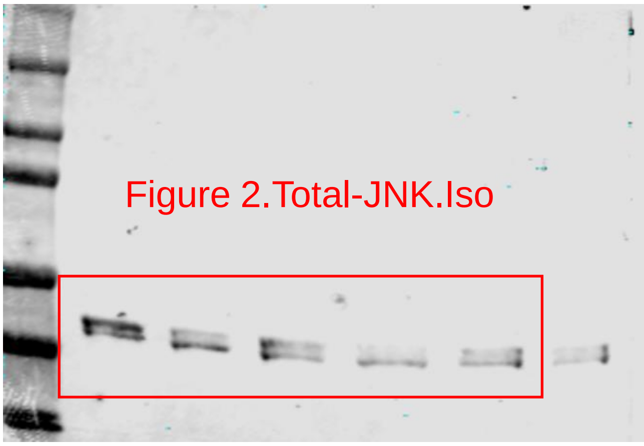

Time (min) 0 5 15 30 60

Isotype

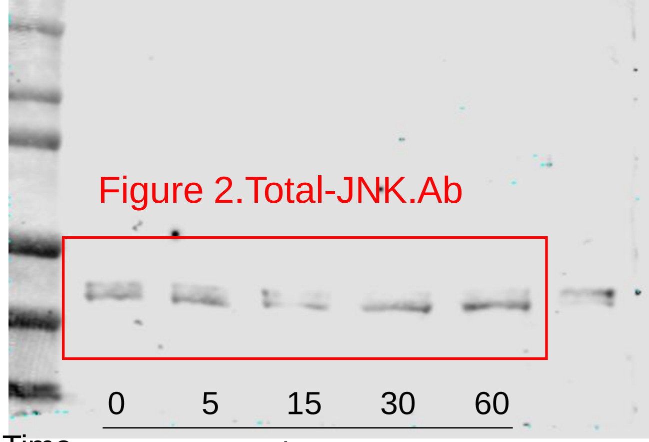

Time (min)

Anti-CD69

Figure 2.Western blot raw

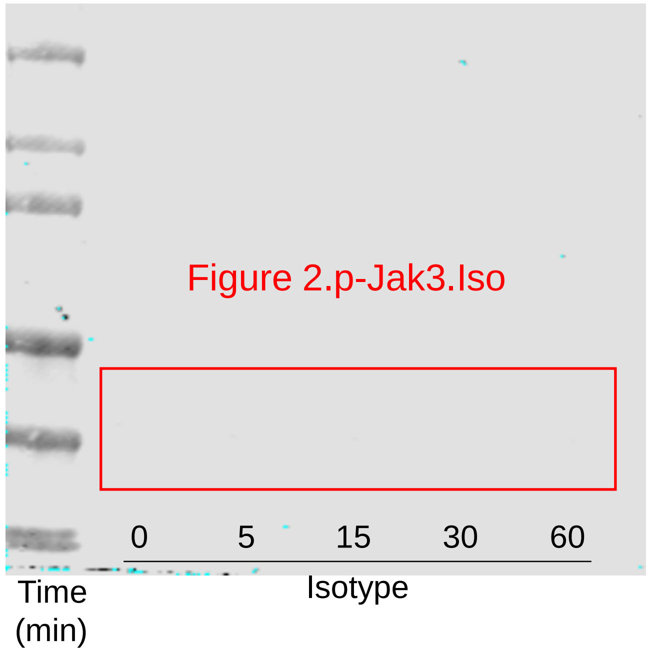

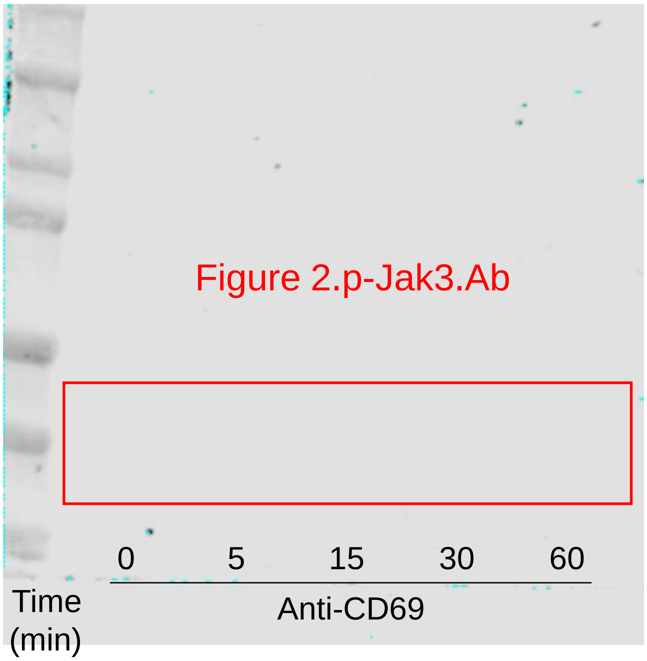

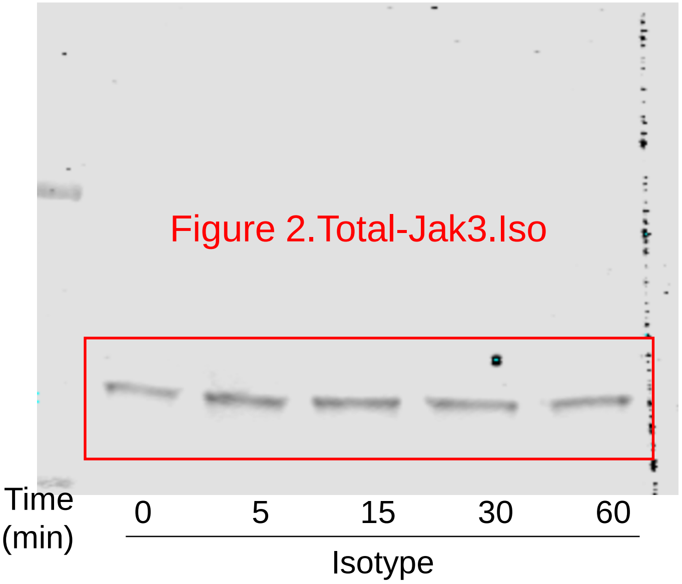

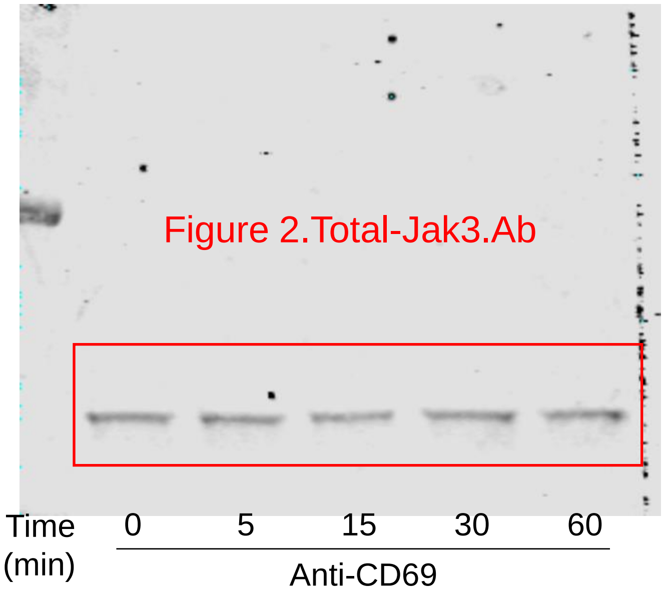

Figure 2.Western blot raw

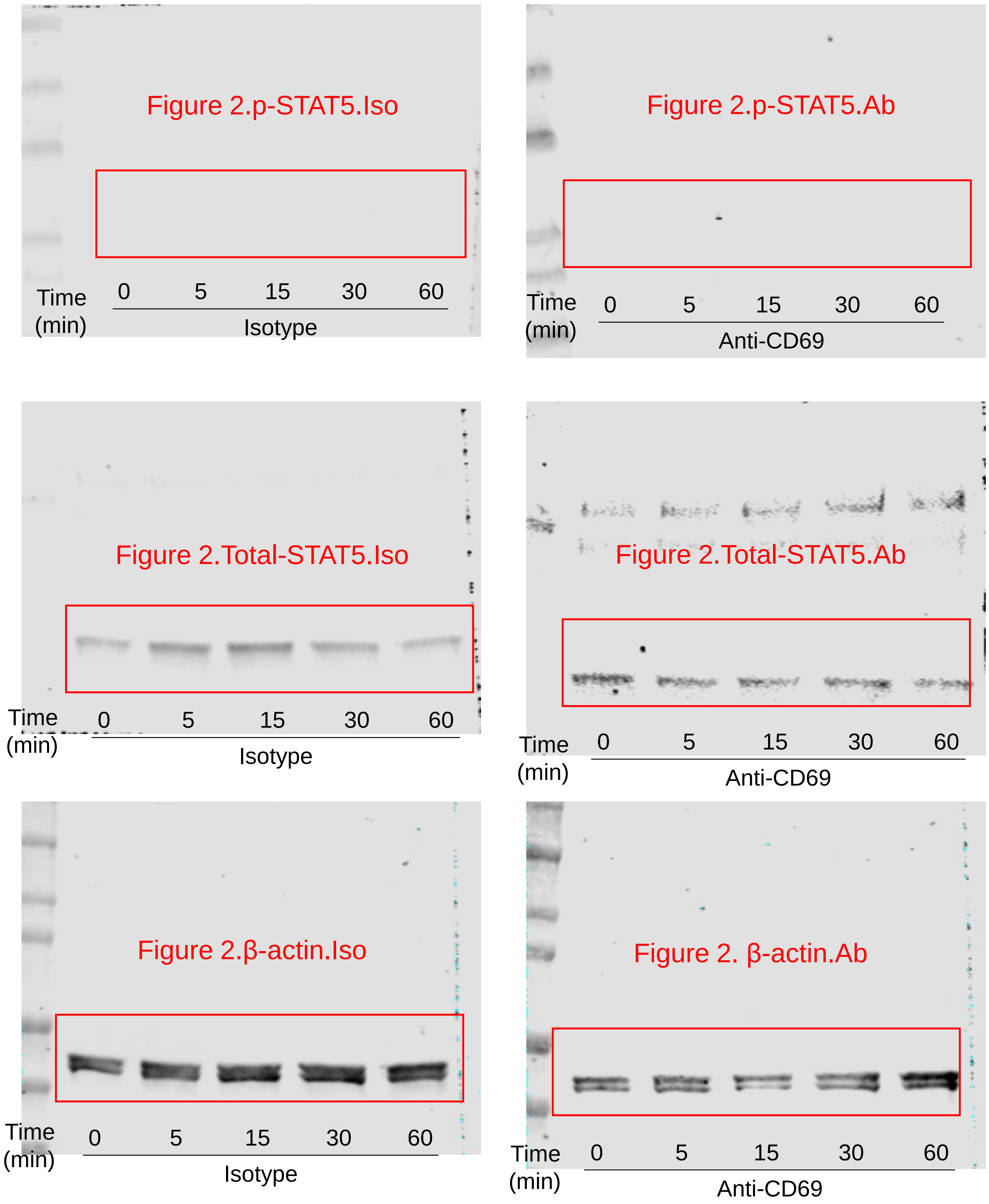

Figure 2.Western blot raw

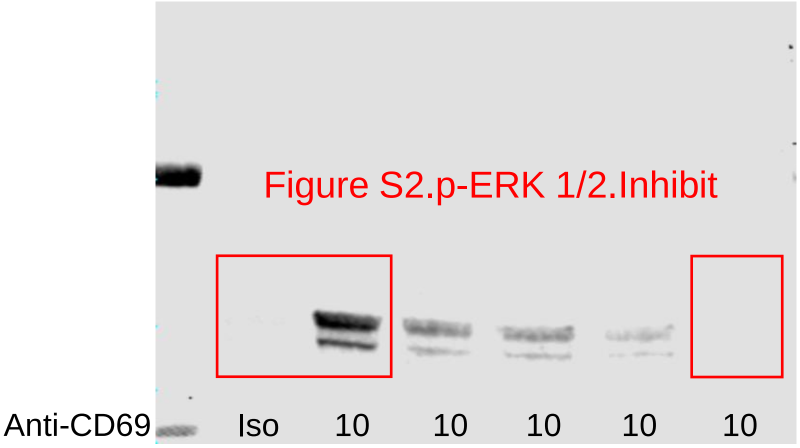

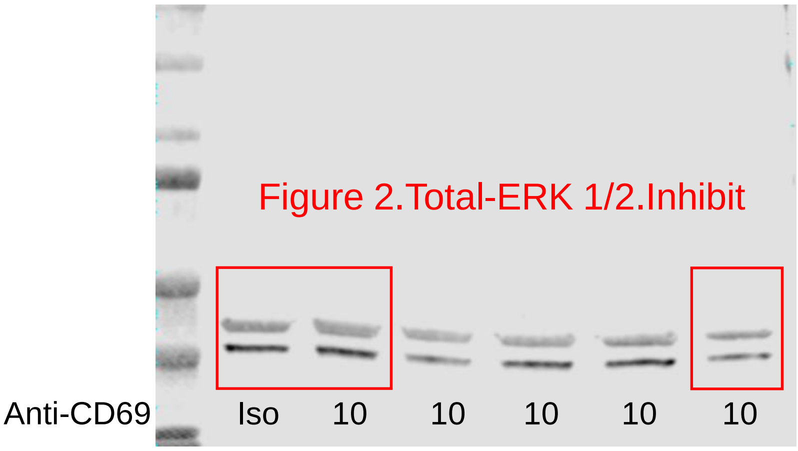

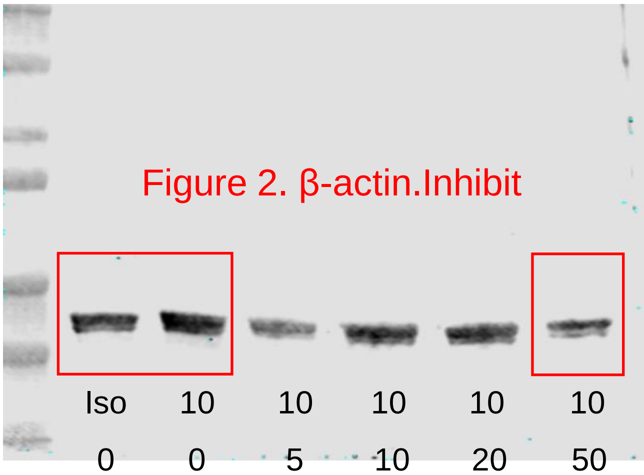

Figure S2.Western blot raw
